# Neurofeedback: potential for abuse and regulatory frameworks in the United States

**DOI:** 10.1098/rstb.2023.0099

**Published:** 2024-10-21

**Authors:** Fiona Furnari, Haesoo Park, Gideon Yaffe, Michelle Hampson

**Affiliations:** ^1^ Yale Law School, New Haven, CT 06511, USA; ^2^ Department of Radiology and Biomedical Imaging, Yale School of Medicine, New Haven, CT 06510, USA; ^3^ Department of Psychiatry, Yale School of Medicine, New Haven, CT 06511, USA; ^4^ Department of Biomedical Engineering, Yale School of Engineering and Applied Science, New Haven, CT 06511, USA; ^5^ Child Study Center, Yale School of Medicine, New Haven, CT 06520, USA

**Keywords:** neurofeedback, law, undue influence, implicit neurofeedback, implicit training, technology regulations

## Abstract

Neurofeedback is a brain-training technique that continues to develop via ongoing innovations, and that has broadening potential impact. Once confined primarily to clinical and research settings, it is increasingly being used in the general population. Such development raises concerns about the current regulatory mechanisms and their adequacy in protecting patterns of economic and political decision-making from the novel technology. As studies have found neurofeedback to change subjects’ preferences and mental associations covertly, there is a possibility it will be abused for political and commercial gains. Current regulatory practices (including disclaimer requirements, unfair and deceptive trade practice statutes and undue influence law) may be avenues from which to regulate neurofeedback influence. They are, however, limited. Regulating neurofeedback will face the line-drawing problem of determining when it induces an unacceptable level of influence. We suggest experiments that will clarify how the parameters of neurofeedback training affect its level of influence. In addition, we assert that the reactive nature of the traditional models of regulation will be inadequate against this and other rapidly transforming technologies. An integrated and proactive regulatory system designed for flexibility must be adopted to protect society in this era of modern technological advancement.

This article is part of the theme issue ‘Neurofeedback: new territories and neurocognitive mechanisms of endogenous neuromodulation’.

## 1. Introduction

A number of recent technological developments have amplified our ability to manipulate human decision-making in powerful, yet subtle, ways. The collection and aggregation of large amounts of personal data makes it possible to tailor advertisements and buying opportunities to individual people; artificial intelligence approaches facilitate such microtargeting and other forms of personalization. And most recently, neurotechnology has been developed that enables the direct monitoring and alteration of mental activity (including sensory perceptions, emotional experiences, states of cognition, etc., all of which can influence decision-making), sometimes without conscious awareness [1–3]. This changing social landscape threatens our economic and political systems in ways that are not currently well understood, much less well regulated.

While different aspects of this complex problem have been explored in prior publications [2,4], the use of neurofeedback to influence decision-making has been given little attention. A number of papers have discussed ethical implications of neurofeedback when used on individuals seeking treatment or self-improvement [3,5–7], but here we are concerned specifically with its potential to be used by some parties to influence the decision-making of others. First, we discuss the literature demonstrating what is known about neurofeedback and its potential for altering human perception and mental function. Second, we describe existing regulatory frameworks in the United States. We discuss how, as neurofeedback is scaled from its limited current use as an individual research or clinical tool to broad usage in the population, current regulatory mechanisms fall short of protecting patterns of economic and political decision-making essential to both regulated capitalism and democracy. Third, we address outstanding questions about the power of neurofeedback and how this varies based on the manner and context in which it is administered. We suggest research that would be helpful for clarifying how and when neurofeedback should be regulated. Finally, we highlight that neurofeedback is only one aspect of a broad and complex social transformation that requires our attention. We advocate for an integrated and proactive approach to addressing neurofeedback’s social impact.

## 2. Neurofeedback and its potential for manipulating mental function

Neurofeedback is a brain-training technique in which individuals are provided with information, in real time, concerning some aspect of their brain function. For instance, they might be presented with a bar on a screen that varies in size with the degree of activation in a given area of their brain, such as, for example, an area associated with craving. The aim is to induce learning that modifies, in a targeted manner, some aspect of their mental function. So, for instance, subjects might be instructed to make the bar smaller when shown pictures of cigarettes so as to reduce their level of craving for nicotine. Participants may be aware of what the feedback signal reflects and what they are learning from it (explicit training, as when they are told what the bar indicates and that the point is to dampen craving) or they may be unaware of what they are learning (implicit training, as when they are told to try to increase or decrease the bar but are told neither what the bar indicates nor how successfully controlling it may impact their mental function). Both forms of neurofeedback have been demonstrated to be effective for inducing targeted learning [8–13].

To provide individuals with information about their changing brain activity patterns, brain activity must be measured. There are a variety of options available for measuring brain activity, but the two most commonly used at present are electroencephalography (EEG), which measures fluctuations of electrical activity in the brain at high temporal resolution (including both ‘brain waves’ and electrical potentials that occur in response to specific events), and functional magnetic resonance imaging (fMRI) which monitors changes in blood oxygenation (or perfusion) that occur subsequent to neural activity changes. EEG is portable and much less expensive than fMRI, and has better temporal resolution. However, fMRI provides rich information about activity fluctuations in specific, localized brain areas. Although EEG neurofeedback has been around longer, fMRI neurofeedback has developed rapidly over the last two decades and has provided strong evidence that neurofeedback can induce effective brain training.

What the growing success of neurofeedback interventions suggests is that there is potential for it to be used to influence individual decision-making in problematic ways. Three key characteristics of neurofeedback learning are relevant to its potential to be used to manipulate individuals: *precision—*it can be used to manipulate precisely specifiable mental activity; *covertness*—it can be used without the affected party’s conscious awareness; and *persistence*—it can be used to induce habits of mental activity that persist for extended periods of time. Additionally, for neurofeedback training to become a tool of broad social influence, it would need to have high *accessibility*—it would need to be useable outside of formal lab environments. We describe below the fMRI neurofeedback literature that has demonstrated a high level of precision, covertness and persistence of neurofeedback learning, and then describe ongoing work to make powerful fMRI neurofeedback protocols more broadly accessible by translating them to portable and accessible modalities, such as EEG.

### (a) Neurofeedback can modulate precise aspects of mental function

A neurofeedback study that reinforced brain patterns in the visual cortex associated with viewing lines of a specific orientation demonstrated that perception of the targeted line orientation could be improved without affecting perception of other line orientations [11]. Specificity has also been demonstrated for higher level aspects of cognition. In one study, for instance, facial preference was manipulated, with some subjects trained towards greater liking, and others towards greater disliking, of faces initially regarded neutrally. The training altered subjects’ preferences for the faces used in the training as intended but did not impact preference for faces that were not used in the training [10]. In some cases, this specificity of fMRI neurofeedback has been so precise as to enable dissociation of mental functions that typically track each other. For example, confidence in ability typically tracks ability; we are usually roughly correct at estimating how good we are at things. However, in a neurofeedback study involving a perceptual task, the subjects’ confidence in their ability to correctly perform the task was manipulated without altering their competence at the task. Neurofeedback was used to make subjects both more and less confident [14]. In short, fMRI neurofeedback has been shown to be capable of altering mental function in precise ways.

### (b) Neurofeedback can induce learning covertly

The studies described above used implicit training: although participants in those studies knew they were getting neurofeedback, they did not know what aspect of their mental function was being trained. Thus, specific learning can be induced via neurofeedback without the individuals involved knowing how their mental function is being altered. Furthermore, neurofeedback may induce effective learning even when participants are unaware they are receiving any information about their brain activity. In a study where participants were misled to believe they were participating in a study of reward processing in the brain, without awareness that the rewards were linked to (and thus reinforcing) specific brain patterns [15], a subset of the participants were found to modulate their brain patterns to amplify rewards, and showed associated modifications in brain network function. These studies are a part of a growing literature reporting successful implicit training, and demonstrating that neurofeedback is a technique with the potential to covertly alter human brain patterns in a targeted manner.

### (c) Neurofeedback can induce persistent changes in brain and mental function

Learning induced by neurofeedback has been shown to persist for weeks [16–18] to months [19–22]. In fact, it appears that the changes in neural and mental function induced by neurofeedback often continue to develop over time after the training has been completed: studies have reported that changes in brain patterns are greater the day after training than immediately after the training [23], and that changes in clinical symptoms induced by neurofeedback continue to grow for weeks to months after the training has been completed [24–26]. Thus, the learning induced by fMRI neurofeedback training not only persists over time, but in many cases, continues to grow.

### (d) Potential for neurofeedback training becoming broadly accessible

Much of the work described above that has demonstrated precision, covertness and persistence of neurofeedback has been based on fMRI neurofeedback. Given the expense and inconvenience associated with fMRI, this form of neurofeedback is unlikely to be used outside of formal lab settings. However, neurofeedback protocols training specific aspects of mental function can be developed via fMRI and then translated to cheaper more accessible imaging modalities, such as EEG. There is great interest in such translation for clinical researchers aiming to create accessible neurofeedback-based treatment options. Feasibility has been demonstrated by successful development of an EEG ‘fingerprint’ of amygdala activity (amygdala is a brain region involved in emotional experiences). In this work, an EEG pattern tracking amygdala activity, dubbed the amygdala EEG fingerprint, or EFP, was identified via simultaneous EEG-fMRI [27,28]. EEG training of the amygdala EFP has been shown to alter amygdala function in healthy and patient populations [27,29] and recently received FDA approval as a PTSD neuromodulation therapy.^1^ Meanwhile, EFPs of other aspects of brain function are under development [30]. Furthermore, EEG is not the only easily disseminated brain-imaging modality: other options include functional near-infrared spectroscopy (fNIRS) and directly implanted electrodes. Notably, major tech companies have been investing heavily in the creation of a wide variety of neurotechnology options as described in detail in Battle for your Brain [2], from EEG-empowered virtual reality set-ups^2^ to earbuds that promise to ‘capture research-grade brain data as you go about your day’.^3^ Of course, it may be that some fMRI neurofeedback paradigms cannot be effectively translated to the lower resolution brain-imaging modalities that enable broad accessibility. However, it has been demonstrated that some can, and the full impact of ongoing work in this area remains to be seen.

In summary, fMRI neurofeedback has been shown to be an effective tool for manipulating human mental processes in a targeted and covert manner and with long-term effects. There are significant efforts underway to make neurofeedback-enabling technologies accessible outside of lab conditions. As academics and tech companies race towards this goal, the potential for abuse of neurofeedback must be recognized and addressed.

## 3. Regulating neurofeedback influence

Neurofeedback technology may soon enable more widespread access to the precise, covert and persistent manipulation of mental functions, inviting the possibility that actors will use neurofeedback for undesirable ends. In this part, we explore legal avenues in the United States for limiting the abuse of technologies of influence. We pay special attention to the potential for abuse in the commercial and political contexts. Whenever new technologies of influence have emerged, actors have used those technologies to exploit consumers and voters. The personalizing of advertisements enabled by social media platforms has become a central tool of both commercial brands and political campaigns [31]. Dark patterns, user interfaces that exploit consumers’ cognitive biases to steer behaviour, are common to both political email blasts [32] and product advertising [4]. Campaigns have been incorporating generative AI into their political advertising ahead of the 2024 US presidential election.^4^ ^5^

In considering regulatory avenues, we uncover two legal frameworks for regulation of influence: threshold-based and feature-based laws.^6^ Threshold-based laws make influence illegal only when it passes a certain threshold. Threshold-based laws may be broad-based human rights laws; for instance, the European Union’s Charter of Fundamental Rights prohibits violations of ‘mental integrity’.^7^ Or, as is the case in the United States, they may be piecemeal domain-specific laws, prohibiting, for instance, the *undue* manipulation of contractual parties^8^ or the *unfair* manipulation of consumers.^9^ Feature-based laws, by contrast, make specific features of influence illegal. As discussed in §2, neurofeedback has some specific influence-enhancing features: precision, covertness and persistence. Some of these features have been regulated in specific contexts. Political advertising, for example, is illegal when it is covert and requires proactive disclosure of influence.^10^

Whether threshold- or feature-based laws adequately address the specific features and risks of neurofeedback influence depends both on how powerful the laws are, whether neurofeedback meets any threshold requirements, and whether regulating specific features of neurofeedback influence is a promising strategy. This part addresses the first of those three questions; the latter are addressed in §4.

The United States currently regulates influence with both feature- and threshold-based laws. While a growing number of countries and international bodies have accepted or proposed laws that cement cognitive liberty as a fundamental right [35], in the United States, existing protections against mental manipulation are inadequate. Scholars and courts have located constitutional protections for the freedom of thought in both the First Amendment and right to privacy [36,37], but these protections are so vague as to lack any teeth. Domain-specific regulations enable some US regulation of political and commercial influence. In the political domain, disclosure requirements are the primary tool for managing undue influence. It is not clear whether disclosure would be helpful for mitigating the impacts of neurofeedback. Furthermore, even if disclosure is helpful, it is only mandated in narrow circumstances that fail to capture the full spectrum of political manipulation that neurofeedback could enable. The commercial context is more promising. Threshold-based consumer protection laws make unlawful unfair and deceptive practices in commerce, such that neurofeedback influence that meets that threshold is illegal. Most encouraging is the common law doctrine of undue influence, which provides that agreements (political, commercial, other) are void when one party has unduly influenced another.

### (a) Threshold-based laws

Threshold-based laws make mental function manipulation unlawful only when that manipulation passes a certain threshold. Not all manipulations of mental functions are illegal. Advertising, for instance, is widely legal. Threshold-based laws draw a line between permissible and impermissible influence. This section primarily focuses on two domain-specific threshold-based legal frameworks in the United States: unfair and deceptive trade practice statutes, which regulate commercial influence, and the common law of undue influence, which limits influence wielded in the course of an agreement. First, however, we may briefly consider a general threshold-based framework for regulating abuse of influence: constitutional protections against mental interference.

US courts generally agree that the right to be free from government manipulation of one’s thoughts is a fundamental one. Yet courts have specified this freedom in terms ranging from a right to be free from ‘unwanted interference’^11^ with one’s mental processes, a low bar, to a right to be free from mind ‘control’,^12^ a much more difficult standard to clear. These differing threshold possibilities have neither been explained nor reconciled [37]. In 1942, Supreme Court Justice Frank Murphy wrote that ‘[f]reedom to think is absolute of its own nature; the most tyrannical government is powerless to control the inward workings of the mind' [36].^13^ As pointed out by neurorights scholars, this is no longer the case [36]. Yet while constitutional protections against mental manipulation are not currently robust in the United States, some neurorights scholars have argued the recognition of freedom of thought may lay groundwork for such rights to be recognized in the future [37].

#### (i) Commercial influence

Unfair and deceptive acts and practices (UDAP) regimes regulate commercial influence in the United States. As the name suggests, these regimes make commercial influence unlawful whenever that influence rises to the level of being *unfair* or *deceptive*. They do so by endowing an agency with the authority to prosecute those practices and to develop further commercial regulation regarding them.

In the United States, the federal agency charged with UDAP regulation is the Federal Trade Commission (FTC). All states additionally have their own UDAP regimes. The FTC is charged with broadly regulating acts in or affecting commerce. Rather than narrowly regulating the interactions between a buyer and seller of a specific product, the FTC may exert its authority to regulate any acts and practices that affect consumers, or persons acting in a commercial capacity. This, for instance, includes discrimination against consumers [38]. As neurofeedback may be used to change preferences [10], it can impact people, even when it is not applied commercially, in their role as consumers. Even neurofeedback exercised to change political preferences may be expected to change consumption habits [39].

The FTC has statutorily defined *unfair* acts and practices as those which 'cause[] or [are] likely to cause substantial injury to consumers which is not reasonably avoidable by consumers themselves and not outweighed by countervailing benefits to consumers or to competition'.^14^ For example, the FTC ordered Epic Games, Inc. to pay $245 million for unfair billing practices^15^ because Epic Games designed the online store for the game Fortnite such that it was exceedingly easy for Fortnite customers to make accidental purchases.^16^ The FTC determined that Epic Games was charging consumers without their express informed consent, causing reasonably unavoidable, substantial net harm ^16^ (p. 13).

The FTC defines *deceptive* acts and practices as material representations, omissions and practices that are likely to mislead a reasonable consumer.^17^ Material representations are those which are likely to impact a consumer’s behaviour with regard to a product. In *Federal Trade Commission v. AMG Capital Management*, the FTC took action against a payday lender, which presented in its loan note a representation of a payment plan—framed as a mandated Truth in Lending Act disclosure—without making obvious to consumers that by default they would be put on an alternative, more expensive plan ([4], p. 83).^18,^ ^19^ While the loan note included complete information in its fine print, it relied on the consumer’s reasonable assumption that the represented plan was the default. For the FTC, this sufficed to show deception: ‘the FTC Act’s consumer-friendly standard does not require only technical accuracy', but that reasonable consumers would not be deceived by representations made ([4], p. 424).

Commercial influence exerted via neurofeedback could satisfy the FTC’s legal standards for unfair and deceptive practices. From their review of the doctrine, Luguri and Strahilevitz conclude that commercial influence (which could potentially be via neurofeedback) may satisfy the substantial injury prong of the unfairness standard whenever consumers make purchases they otherwise would not ([4], p. 88). As in Epic Games’s case, many small purchases by consumers may add up to substantial harm.^16^ It should additionally fulfill the reasonably unavoidable prong in many cases. As noted earlier, researchers have shown that neurofeedback can alter or bias mental processes without participant awareness that they are being influenced [8,10,19]. Such covert neurofeedback cannot be reasonably avoided. Even when consumers are aware that they are undergoing neurofeedback training, they may not have adequate understanding for an ‘informed exercise of consumer choice’.^20^ Participants in numerous neurofeedback studies have reported no understanding of the mechanism or target of the neurofeedback training despite awareness that they were undergoing training [8,10,19]. The FTC has explained that ‘[w]hether some consequence is "reasonably avoidable" depends, not just on whether people know the physical steps to take in order to prevent it, but also on whether they understand the necessity of actually taking those steps’ (p. 1066),^21^ which depends critically on knowledge regarding the nature of the consequence. Thus neurofeedback is not reasonably avoidable when the aspect of mental function trained is not disclosed.

Neurofeedback influence may also be deceptive when it leverages mistaken consumer expectations. The FTC has labeled practices that exploit consumers’ reasonable expectations to be deceptive (pp. 84–85) [4]. When consumers expect that neurofeedback influence works by a particular mechanism and that expectation changes consumer behaviour, that consumer may have been materially deceived. We primarily focus in this article on innovations in neurofeedback technology that, covert, precise and persistent in effect, do not *need* to leverage consumer expectations to exercise influence and thus are more likely to be unfair than deceptive. But some scholars have critiqued less sophisticated neurofeedback technology, such as some EEG devices, as deceptive given they may actually exercise influence via the placebo effect [40].

The potential of neurofeedback to exercise influence by genuinely reshaping preferences may make it especially difficult to regulate. Compared with Fortnite players, consumers influenced via neurofeedback end up not with unwanted products, but with desired products that they would not have wanted but for that influence. While neurofeedback influence hinders consumer choice, it may be more difficult to prove substantial injury to consumers in the case of neurofeedback influence compared with, say, dark patterns. Yet the FTC has fined companies when those companies delivered products customers desired, but nevertheless misled customers into those purchases. For example, the FTC took an action against Office Depot after the company misled consumers as to how it was generating personalized recommendations for malware removal software despite the fact that consumers received software meeting their requirements [4].^22^

#### (ii) Undue influence

Undue influence is a common law defence against the formation of contracts via manipulative means. If a person or platform uses neurofeedback to encourage another to buy a good or otherwise enter an agreement, that person or platform may be unduly influencing the other party, such that any agreement formed is invalid.^23^ Per the Second Restatement of Contracts,^24^ to reach the threshold of undue influence, two elements must be met: (i) the unfair persuasion of a target by an influencer, where (ii) the influencer dominates the target or the influencer and target are in a relationship, such that the target believes that the influencer is looking out for their best interest.

In determining the first element, unfair persuasion, ‘[t]he ultimate question is whether the result was produced by means that seriously impaired the free and competent exercise of judgment. Such factors as the unfairness of the resulting bargain, the unavailability of independent advice, and the susceptibility of the person persuaded are circumstances to be taken into account in determining whether there was unfair persuasion, but they are not in themselves controlling’ (§ 177 cmt. c.).

The second element involves either domination or a relationship. This could be one contractual party, A, dominating or being in a relationship with the other contractual party, B, or it could be one third party, C dominating or being in a relationship with B on behalf of A [40].

California courts have found domination to have two requirements: excessive pressure applied by the influencer and undue susceptibility of the target. In a paradigm case, *Odorizzi v. Bloomfield School District*, the California Second District Court of Appeals found that a teacher had been unduly influenced to resign after school officials used a ‘high-pressure carrot-and-stick technique’ (p. 135) to convince him to resign immediately after the teacher had spent 40 sleepless hours in police custody [4].^25^ The court held that one party dominates another when it applies ‘high pressure’ to the other that ‘works on mental, moral, or emotional weakness to such an extent that it approaches the boundaries of coercion’ ([4], p. 130).

Historically, undue influence doctrine has been centered on elderly or ill individuals writing wills under pressure [42], where the relationship between the two parties may be trustee and beneficiary [43] or caretaker and client. But some courts have construed relationships more broadly in the context of undue influence law, as Luguri & Strahilevitz observe ([4], p.94). Generally, courts have found a relationship in cases where the targeted party has reason to rely on the influencer [43]. Courts have found that friendships in which one is an attorney^26^ and doctor–patient treatment relationships^27^ are adequate to fulfill the second element. And users may reasonably depend on digital platforms, too, such that the consumer–platform relationship fulfills the relationship requirement. Luguri & Strahilevitz argue that if we adopt Jack Balkin [44] and Jonathan Zittrain’s^28^ proposal that major digital platforms owe fiduciary duties to their consumers, unfair persuasion by a platform of its users would be undue influence ([2], p.95). This is because the law presumes undue influence when a fiduciary benefits from a transaction with a client to whom it owes a duty [4].^29^

Neurofeedback may be used to exert undue influence. Depending on the qualities of the training platform, a neurofeedback provider may be in a treatment relationship with users or otherwise owe users fiduciary duties. Further, any influence that compromises the exercise of free judgment is considered unfairly persuasive. Neurofeedback influence may have the potential to compromise free judgment. After all, neurofeedback exerts subconscious conditioning, and so bypasses an agent’s conscious reasoning processes. Of course, legally, not every subconscious influence compromises free judgment (nostalgic television advertisements are not unlawful); §4 suggests further research to better understand neurofeedback influence and when and whether it is undue. However, provided that neurofeedback processes do compromise free judgment—which seems likely, particularly in the case of implicit neurofeedback—neurofeedback influence is arguably intrinsically unfairly persuasive.

Luguri & Strahilevitz argue that undue influence doctrine shows the most promise as a legal framework for regulating cognitive exploitation via dark patterns (p. 94) [4], p. 94); undue influence doctrine likewise may provide the best pathway towards curtailing neurofeedback influence, even beyond contract law. Indeed, Montana’s voter intimidation statute prohibits the ‘use or threat’ of ‘undue influence against any person’ to induce that person to vote or refrain from voting for a candidate, political party or ballot issue.^30^ In *Burson v. Freeman*, the Supreme Court of the United States recognizes a ‘compelling interest in protecting voters from confusion and undue influence’ (p. 199).^31^ It is possible, therefore, that the best way to regulate neurofeedback will be through the application of undue influence doctrine.

### (b) Feature-based laws

Political and election influence regulation is significantly limited by the need to avoid placing unconstitutional restrictions on freedom of speech^32^—the First Amendment of the United States Constitution is understood to protect political speech more robustly than any other sort. Mandating disclosure limits manipulation by eliminating influence-enhancing features of speech rather than limiting speech itself.

Disclosure requirements extend to paid political advertisements, including digital advertisements.^33^ Political advertisements for federal candidates must feature a ‘clear and conspicuous’ disclaimer,^34^ including who paid for the advertisement and whether a specific candidate or committee authorized the advertisement.^35^ Every state has some sort of disclaimer law requiring an individual or committee behind an advertisement to publish who paid for it—‘This ad was paid for by …’.^36^ Some states include further requirements—Washington State, for instance, requires advertising hosts, such as Facebook or The New York Times, to make available upon request the demographic targets of an advertisement.^37^

Mandatory disclaimers have long been justified on the basis that they help voters make ‘an informed choice in the political marketplace’ (p. 367).^38^ When an individual recognizes a disclaimer, that disclaimer enables them to research or recognize the committee or individual sponsoring the advertisement. In the case of digital ads, disclaimers additionally reveal that a piece of digital content *is* an advertisement, rather than just another social media post. When people detect that a piece of content aims to persuade them, they may employ cognitive coping strategies to resist persuasion [45–47]. Coping strategies, such as counterarguing, reduce the effectiveness of persuasion [48].

It is unclear how effective disclaimers are in mitigating the influence of digital political ads. (Below, we suggest a way of gathering data about its capacity to mitigate the influence of neurofeedback.) Users consistently fail to recognize commercial and political advertisements are advertisements even with disclaimers. Studies have shown only about 10% of participants noticing that an article with a sponsored content disclaimer was sponsored content on a news website [46], and about 20% of participants recalling a political advertisement in a Facebook feed, despite that advertisement including Facebook’s political advertising disclaimer [49]. In addition to being difficult to notice, many people do not understand the implications of a disclaimer. In one study, half of participants wrongly believed that political advertisements must be factchecked before they are aired, and two in five wrongly believed that the law requires political advertisements to be true and not misleading [50]. Disclaimer requirements also generally only apply to paid election influence [51]. If a neurofeedback platform decides to exercise influence over an election without being paid to do so, they may do so without issuing a disclaimer. This leaves open the possibility of unilateral campaigns by platform owners [52].

Perhaps most importantly, disclaimer requirements federally and in most states apply narrowly to election influence, rather than broader political influence. If an advocacy group paid a neurofeedback platform to influence consumers’ attitudes towards crime, education or reproductive rights, even in the days leading up to an election, campaign finance regulations would provide little recourse. Federal regulations require disclaimers on any public communication,^33^ mass email or website made by a political committee^35^; any public communication by any person that expressly advocates for the election or defeat of a clearly identified candidate^35^; any public communication that solicits a contribution^35^ and any broadcast, cable or satellite communication that refers to a clearly identified federal candidate, is targeted to the relevant electorate, and is publicly distributed near an election date.^39^ If an advocacy group or individual (not a political committee) creates an advertisement centered on an issue (not referring to a candidate) that is informing the public (not asking for a contribution) none of the foregoing disclosure requirements are likely to apply.^40^

### (c) Improving regulatory protection

Abuses of neurofeedback influence are not easily reparable; prevention is preferred. Given neurofeedback is capable of genuinely changing preferences [5], perceptions [6] and mental associations [14], individuals who have been influenced to buy a specific product may not feel harmed by that purchase. They may further be unlikely to file a complaint with the FTC regarding that product, or to raise a claim of undue influence in court—even more so if the influence was covert. Furthermore, neurofeedback influence, in addition to being covert and precise, is persistent; preference changes may last for at least months. Legal paths that require sorting out after the fact whether the influence in question was undue, or the manipulation unfair, come too late.

Prevention may occur through the use of feature-based laws, such as disclosure requirements. Prevention may also be achievable when thresholds are clear and technological capabilities are thoroughly assessed with regard to whether they meet those thresholds. Meeting the thresholds of undue influence, unfair persuasion and ‘unwarranted infringement into one’s mental processes’ depends in part on *magnitude* of influence. In the following section, we propose experiments to help determine the magnitude of neurofeedback influence, and to help elucidate whether disclosures may indeed mitigate potential neurofeedback influence.

We additionally urge a focus on new legislation that clarifies when mental manipulation becomes undue. Pursuing post-injury litigation in an attempt to get courts to recognize that neurofeedback influence is undue influence or that freedom of thought does constitutionally protect against mental manipulation is worthwhile. But we have an opportunity to do better. We could advocate, for instance, for legislation that eliminates the requirement of a relationship under undue influence law in cases of technology-mediated influence, so that ‘extraordinary force’ of influence is sufficient. While higher influence neurofeedback technologies are on the path towards wider accessibility, they are not yet broadly accessible. We should take this opportunity to better understand the risks, magnitude and character of neurofeedback influence, and pursue legislation that best prevents those risks from coming to fruition.

## 4. Directions for the investigation of neurofeedback scenarios that amplify or reduce influence

The magnitude of neurofeedback influence, along with other contextual issues, determines the degree to which it undermines freedom, which in turn affects the degree to which it ought to be considered undue or unfair. Thus, decisions regarding whether influence is undue or unfair require knowledge regarding the magnitude of influence and more information is needed to better understand the parameters that govern magnitude of neurofeedback influence. For example, if implicit neurofeedback is twice as impactful as explicit neurofeedback then we should regulate it more stringently accordingly, and whether the magnitude of influence increases or plateaus with repetition of training events will affect whether greater oversight is necessary with more training. Here we propose studies that would inform us regarding the magnitude of neurofeedback influence in different contexts.

### (a) Impact of disclosure on neurofeedback learning

As noted in §3b, due to free speech protections, the US government by and large cannot currently constrain efforts to use speech to influence political decision-making farther than mandating disclosure; an ad supporting a vote for a candidate must include a message saying that the ad is paid for by the candidate. Disclosure regulations have several functions, including transparency and other requirements of a democratic state. They are also in part based on the belief that disclosure gives an individual the capacity to override an influence once they are aware it is being exerted. However, it is not known if this is true in the context of neurofeedback: it is an empirical question whether disclosure makes it possible for people subjected to implicit neurofeedback to resist its influence. An experiment investigating this question would be helpful. Here we describe a study specifically aimed at determining whether disclosure can mitigate neurofeedback influence on facial preferences. We distinguish between disclosure provided before training and disclosure provided after training, as these may have different impacts on training efficacy.

The proposed study would build on work showing implicit neurofeedback can be used to modify facial preferences. This finding raises concerns about political influence, given the literature demonstrating that favourable impressions of how candidates look may have a substantial impact on voting behaviour [53]. As political influence is typically regulated by requiring disclosure, it is important to know whether disclosure can effectively mitigate the impact of neurofeedback.

This question could be addressed by replicating the study where neurofeedback was used to influence facial preference, but expanding it into a larger trial with three arms: one which receives no disclosure regarding how the neurofeedback is aiming to change facial preference, one group that receives pre-neurofeedback disclosure, and one that receives a disclosure regarding the purpose of the training after the training occurs, but before the self-reporting of facial preference (post-neurofeedback disclosure). Stimuli that are initially ranked neutrally for each subject will be divided into three sets: one that will be trained up in preference, one that will be trained down, and one that will not be trained. After training, participants will again rate their level of preference for the faces and will be exposed to the faces again in the scanner to determine if neural markers of ‘liking’ have been shifted. The difference in neural markers and preference ratings for faces between the ‘up’ and ‘down’ stimuli will be compared across groups to see if disclosure modified the influence of neurofeedback on facial preference. Participants will also be asked after study completion how they feel about having their facial preferences altered to explore whether this variable modulates the impact of disclosure (given that those who dislike having their facial preferences influenced may resist the effects of training, while others may not).

The primary aim of this proposed experiment is to provide data that could rationally guide policymaking about neurofeedback treatments aimed at influencing voting behaviour. As discussed above, there are reasons to think that disclosure requirements may not always accomplish the aim of guiding voters to make rational decisions in the face of advertising. However, if the effect of neurofeedback is significantly dampened by disclosure, that will suggest that applying laws requiring disclosure to those using neurofeedback may be an effective approach to protecting society from neurofeedback abuse. Conversely, if neurofeedback impact is magnified by disclosure, that would provide reason for policymakers to discourage disclosure of neurofeedback training. If neurofeedback is unaffected by disclosure, this will highlight the inadequacy of disclosure as a regulatory tool for protecting society from neurofeedback-induced political disruption. To know what approach is best, policymakers will be aided by data of the sort that our proposed experiment would supply.

### (b) Mapping how amount of training impacts neurofeedback learning

A distinctive aspect of neurofeedback learning relative to traditional forms of influence seen in the commercial and political spheres is that it involves a systematic application of influence: neurofeedback involves many learning events (typically hundreds to thousands) all engineered to influence an individual in the same manner. Although the influence of neurofeedback (and other feedback learning-based interventions) probably scales with the amount of repetitive exposure received, it is not known exactly how this scaling works. For example, is there some optimal level of training after which little additional learning occurs? If so, it should be possible to identify the maximal impact of a given type of training regardless of how long and extensively it is applied. Alternately, if the relationship between training and learning is essentially linear, with more feedback yielding greater influence in a steadily increasing manner indefinitely, extensive training may be capable of inducing extremely powerful effects. In this case, special attention should be given to situations where extensive training is possible, such as is feasible with portable devices used regularly for long periods of time.

These kinds of questions can be addressed by neurofeedback studies that randomize subjects to receive different ‘doses’ of neurofeedback and measure the resulting influence on mental function. Depending on the context, it may also be important to follow participants for a period of time after the training in order to distinguish immediate effects on mental function and behaviour from longer term effects. These may be markedly different. For example, clinical impacts of neurofeedback have been reported to grow over time after the training is complete [25]. Thus, in any context where longer term impacts are socially relevant, it is critical to measure them empirically.

Ideally, studies of this nature will be conducted on many different neurofeedback paradigms to explore whether consistent patterns emerge that can be generalized. However, there are many different forms of neurofeedback (targeting different aspects of brain function and using different feedback interfaces). Thus this kind of work, although important, is unlikely to ever be ‘complete’, and will never provide more than a heuristic estimate of potential influence (albeit one that hopefully improves steadily as more data are acquired).

### (c) Proceeding in parallel with regulatory development

The pace of technological development and its potential for rapid transformation of society are rendering traditional models of regulation, that require collection of extensive data prior to implementing policies, inadequate. Decisions must be made rapidly and regulatory systems designed for flexibility such that new scientific knowledge can quickly be translated into improvements in the regulatory framework. Given this, these decisions require close collaboration and communication between policymakers and neuroscientists. This is an international challenge: thus it may be helpful to examine the different approaches adopted across the globe and their comparative strengths and weaknesses [54].

## 5. Conclusion

Changes in legal regulation that are responsive to technological development are notoriously slow. Often, a new technology is being used in a problematic way for many years before laws are passed to curb the problem.^41^ As discussed above, one reason for this is the slow nature of traditional regulatory approaches. However, another important reason is that neither the policymakers nor the technological innovators anticipate the possibility of abuse; there can be no regulatory steps taken until it is recognized that they are needed. Our goal here has been to anticipate the possibility for abuse of neurofeedback technology in influencing voting and consumer behaviour. Additional knowledge will be helpful to know how damaging, if at all, different forms of neurofeedback influence can be. While we have identified two different things about which more needs to be known—(i) whether disclosure of the nature and purpose of neurofeedback training changes its influence and (ii) how neurofeedback influence scales with the amount of training—we are open to the possibility that other questions, perhaps more pressing questions, need to be answered to guide the optimization of legal and regulatory frameworks. However, more thought needs to be given to this issue, and soon, given the pace at which neurofeedback is developing.

Neurofeedback advances are but one part of a broader technological transformation that will have widespread social, ethical, and legal consequences. Just as we propose considering the extent to which disclosure mitigates neurofeedback influence, urgent questions continue to emerge about the potential of disclosure as a tool for mitigating technological influence and misinformation more broadly. In September 2023, Google announced that all election advertisements on Google or YouTube’s platforms must have a clear and conspicuous disclaimer whenever they contain AI-generated content.^42^ Similarly, just as we propose that the existing legal patchwork is insufficient for regulating advancements in neurofeedback, legislators and leading AI researchers are advocating that existing laws and agencies are inadequate for regulating advancements in generative AI.^43^ Finally, in the realm of neurotechnology, increasing ease of gathering neurodata has contributed to growing calls for neurorights, brain data privacy and the recognition of cognitive liberty [2]. These concerns are all the more pressing given the likelihood that these different forms of technology are unlikely to be used solely in isolation, but more probably will be combined so as to amplify their influence in complex ways.

To regulate reactively, based upon the technological risks apparent today threatens to create a new inadequate legal patchwork: laws set up to tackle discrete technological mechanisms rather than technological integration and broad-based change. Technological progress is integrated and rapid; our regulatory response ought to be as well.

## Data Availability

This article has no additional data.

## Footnotes

^1^

https://www.mobihealthnews.com/news/graymatters-health-receives-fda-510k-ptsd-neuromodulation-therapy

^2^

https://www.cnet.com/tech/computing/i-wore-the-future-of-brain-connected-arvr

^3^

https://www.emotiv.com/product/mn8-2-channel-eeg-earbuds

^4^

https://farid.berkeley.edu/deepfakes2024election

^5^

https://thehill.com/opinion/campaign/4151633-yes-we-should-regulate-ai-generated-political-ads-but-dont-stop-there

^6^
The division between threshold-based laws, which generally require judges to deploy moral concepts such as unfairness or undueness, and feature-based laws, which set harder limits on conduct, is similar to other frameworks for dividing up our legal obligations, such as Ronald Dworkin’s distinction between principles and rules [33] and the common jurisprudential divide between standards and rules [34].

^7^
European Union Charter of Fundamental Rights, 2000 O.J. (C364).

^8^
Restatement (Second) of Contracts § 177 (1981).

^9^
15 U.S.C. § 45(n).

^10^
11 C.F.R. § 110.11 (2023).

^11^

*Stanley v. Georgia*, 394 U.S. 557, 565 (1969).

^12^

*Rennie v. Klein*, 462 F. Supp. 1131, 1144 (D.N.J. 1978).

^13^

*Jones v. Opelika*, 316 U.S. 584, 618 (1942).

^14^
U.S.C. § 45(n). The FTC may additionally take public policy into account when evaluating unfairness, though it may not be the primary basis for an enforcement action.

^15^

https://www.ftc.gov/news-events/news/press-releases/2022/12/fortnite-video-game-maker-epic-games-pay-more-half-billion-dollars-over-ftc-allegations

^16^
Epic Games, Inc., FTC Docket No. C-4790 (13 Mar. 2023).

^17^
John D. Dingell, FTC Policy Statement on Deception (14 Oct. 1983), https://www.ftc.gov/system/files/documents/public_statements/410531/831014deceptionstmt.pdf

^18^
See Fed. Trade Comm'n v. AMG Cap. Mgmt., LLC, 910 F.3d 417, 424 (9th Cir. 2018), rev'd on other grounds and remanded sub nom. AMG Cap. Mgmt., LLC v. Fed. Trade Comm'n, 141 S. Ct. 1341 (2021), and vacated sub nom. Fed. Trade Comm'n v. AMG Cap. Mgmt., LLC, 998 F.3d 897 (9th Cir. 2021).

^19^
In re Cliffdale Assocs., Inc., 103 F.T.C. 110, 1984 WL 565319, *37 (1984) (applying and clarifying legal standard for deception).

^20^
Wendell H. Ford, FTC Policy Statement on Fairness (17 Dec. 1980), https://www.ftc.gov/legal library/browse/ftc-policy-statement-unfairness (introducing and elucidating the FTC’s definition of unfair acts and practices).

^21^
In re International Harvester Co., 104 FTC 949, 1066 (1984).

^22^

*Fed. Trade Comm’n v. Office Depot*, Complaint for Permanent Injunction and Other Equitable Relief, Case No. 9 19-cv-80431 (S.D. Fla. 27 March 2019).

^23^
Undue influence may also be exerted via a third party to the contract. For example, if a contractual party A is benefitting from a third party C’s undue influence over contractual party B, then A and B’s contract may be invalid due to third-party undue influence provided that A either has reason to know about C’s imposition of influence or where A is otherwise transacting in bad faith [41].

^24^
Restatement (Second) of Contracts § 177 (1981). The Restatement is a legal treatise which summarizes common law developments across state and federal courts.

^25^

*Odorizzi v. Bloomfield Sch. Dist.*, 246 Cal. App. 2d 123, 135 (Ct. App. 1966).

^26^

*Goldman v. Bequai*, 19 F.3d 666, 669 (D.C. Cir. 1994) (holding that a relationship between a widow and her long time friend, where the friend is an attorney, suffices for the relationship component of undue influence).

^27^

*Gerimonte v. Case*, 712 P.2d 876 (Wash. App. 1986) (holding that a chiropractor’s influence over a patient constitutes undue influence even though the urging was similar to that of an everyday business transaction).

^28^

https://www.theatlantic.com/technology/archive/2016/10/information-fiduciary/502346

^29^
Consider, for example, the case of *Matlock v. Simpson*, 902 S.W.2d 385 (Tenn. 1995).

^30^
Mont. Code Ann. § 13-35-218 (2021).

^31^
Burson v. Freeman, 504 U.S. 191, 199 (1992).

^32^
Regulations restricting or banning speech are strictly limited by the U.S. Constitution. The U.S. Supreme Court allows time-place-or-manner restrictions of speech provided they are content neutral, narrowly tailored to serve a significant government interest, and leave open alternative channels of communication (*Ward v. Rock Against Racism*, 491 U.S. 781, 791 (1989)). Restrictions prohibiting advertising in the polling place, for instance, have been upheld (*Burson v. Freeman*, 504 U.S. 191, 199 (1992)). To limit some forms of neurofeedback influence—such as political influence—and not others would fail to be content neutral.

^33^
11 C.F.R. § 110.11(c)5 was revised on 1 March 2023 to clarify how disclaimer requirements apply to internet communications. https://www.fec.gov/updates/commission-adopts-final-rule-internet-communications-disclaimers-and-definition-public-communication/

^34^
11 C.F.R. § 110.11(c)(3) (2023).

^35^
11 C.F.R. § 110.11 (2023).

^36^
For a survey of state laws providing for disclaimers in the case of digital advertising, see the National Conference of State Legislators, Survey of State Political Advertisement Disclaimers (14 Mar. 2023), https://www.ncsl.org/elections-and-campaigns/disclaimers-on-political-advertisements

^37^
Wash. Admin. Code § 390-18-050. Facebook does not currently accept any ads for Washington State candidates, as they do not wish to comply with these disclosure requirements.

^38^

*Citizens United v. Fed. Election Comm'n*, 558 U.S. 310, 367 (2010).

^39^
11 C.F.R. § 100.29 (2023).

^40^

*Fed. Election Comm'n v. Wisconsin Right To Life, Inc.*, 551 U.S. 449, 469–70 (2007) (holding that political advertisements that ‘focus on a legislative issue’ and ‘lack[] indicia of express advocacy’ may not be regulated under the First Amendment).

^41^

https://www.nytimes.com/2023/08/24/upshot/artificial-intelligence-regulation.html?campaign_id=9&emc=edit_nn_20230824&instance_id=100758&nl=the-morning&regi_id=91509782&segment_id=142776&te=1&user_id=a6684a476f35f1f2fc2ce863f0542b61

^42^

https://www.reuters.com/technology/google-make-disclosure-ai-generated-content-mandatory-election-advertisers-2023-09-06

^43^

https://techpolicy.press/transcript-senate-judiciary-subcommittee-hearing-on-oversight-of-ai
